# KNUST aging and human immunodeficiency virus outcomes—Study protocol

**DOI:** 10.1371/journal.pone.0307719

**Published:** 2024-08-16

**Authors:** Alex Werekuu, Nana Kwame Ayisi-Boateng, Nadia Tagoe, Douglas Aninng Opoku, Bernard Barnie, Godfred Kumi Twumasi, Yaa Twumwaa Boadu, Kaku So-Armah, Phyllis Tawiah

**Affiliations:** 1 Department of General Internal Medicine, Boston Medical Center, Boston, MA, United States of America; 2 Department of Medicine, School of Medicine and Dentistry, Kwame Nkrumah University of Science and Technology, Kumasi, Ghana; 3 Office of Grants and Research, Kwame Nkrumah University of Science and Technology, Kumasi, Ghana; 4 School of Public Health, Kwame Nkrumah University of Science and Technology, Kumasi, Ghana; 5 Metro Health Services, Centre for Aging, Kumasi, Ghana; PLOS: Public Library of Science, UNITED KINGDOM OF GREAT BRITAIN AND NORTHERN IRELAND

## Abstract

**Introduction:**

Globally, 7 million people with HIV (PWH) aged over 50 years exist. 5 million of them live in sub-Saharan Africa, the epicenter of the HIV epidemic. In Ghana, every 1 in 6 PWH is aged over 50 years. However, access to geriatric health care is grossly limited in Ghana and the sub-Saharan Africa region. This has resulted in a lack of focus on geriatric syndromes, a multi-factorial clinical condition common in older PWH, that do not fit discrete disease categories. Consequently, this gap threatens the life expectancy for aging PWH, necessitating the need to promptly fill it. The KNUST Aging and HIV Outcomes (KAHO) study will help identify priorities and opportunities for developing an effective integrated model of HIV and geriatric healthcare in Ghana.

**Methods and analysis:**

The KAHO study will recruit 151 PWH aged 50 years and older at the Infectious Disease Unit (IDU) of the University Hospital, Kwame Nkrumah University of Science and Technology (KNUST). The study will be conducted over a 2-year period and participants will be seen at months 0, 6 and 12. Participants at each visit will be taken through assessments and questionnaires on geriatric health, cognition, social vulnerability, HIV-related conditions and they will provide biospecimens for laboratory testing. We will also conduct semi-structured qualitative interviews of PWH, healthcare providers, policy makers and study research assistants. Quantitative data will be analyzed using one sample proportion test and linear regression models appropriately. The Levesque’s framework will be used as a guide to analyze qualitative data.

## Introduction

Globally, the number of people aged 80 years and over is expected to triple to 426 million between 2019 and 2050; a positive change largely driven by aging in African countries [1]. Due to life-extending HIV therapy, there are an estimated 7 million people with HIV (PWH) aged over 50 years worldwide; 5 million of them live in sub-Saharan Africa, the epicenter of the HIV epidemic [2]. Of the approximately 312,000 PWH in Ghana in 2019, almost 1 in 6 are aged over 50 years [3]. Efforts underway in the region leverage on the general care systems to diagnose and manage chronic diseases of aging. A gap in these efforts is the lack of focus on geriatric syndromes–multi-factorial clinical conditions, common in older adults, that do not fit discrete disease categories [4,5]. Geriatric syndromes (e.g., frailty, multimorbidity) [4,6,7] and discrete chronic diseases of aging such as, hypertension and other cardiovascular diseases, threaten these gains in life expectancy for aging PWH.

Frailty is a geriatric syndrome marked by decreased physiological and functional reserve that increases vulnerability to adverse outcomes like hospital stays, poor vaccine response, functional decline and death [8–10]. Frailty prevalence among PWH ranges from 5–29% and HIV is an independent risk factor for frailty [11,12]. Prevalence among PWH with <10 years treatment duration is approximately 15% [13]. Multimorbidity refers to multiple, potentially interacting physical and mental health conditions and is common in PWH [7,14]. European forecasts suggest that multimorbidity among PWH will increase to 84% by 2030 with over half using multiple medications and cardiovascular disease contributing the greatest burden [7,15].

The threats of geriatric syndrome are particularly troubling in Ghana and sub-Saharan African countries where access to geriatric care and chronic disease management is limited [16–18]. Filling this gap, particularly in the sub-Saharan Africa region, is the next barrier to extending quantity of life and preserving quality of life for aging PWH [7,19–22].

To help fill this gap, our long-term goal is to provide comprehensive care for geriatric syndromes and diseases of aging for older PWH [23]. To achieve this, we are undertaking the KNUST Aging and HIV Outcomes (KAHO) study to help identify priorities and opportunities for building an effective integrated model of HIV and geriatric healthcare in Ghana. This integrated model will extend the gains in healthy life expectancy for Ghanaians and other Africans in similar settings who are aging with HIV.

### Specific aims

The primary outcome for this study is to characterize geriatric syndrome (e.g., frailty, multimorbidity) and discrete diseases of aging among older PWH. Secondary outcomes include; obtaining estimates of under-diagnosis and management of discrete cardiometabolic diseases in older PWH; identifying barriers and facilitators to providing effective, patient centered healthcare to aging PWH; and research capacity building among study staff and other healthcare workers.

#### Aim 1: Characterize frailty and multimorbidity in older PWH in care at the University Hospital, Kwame Nkrumah University of Science and Technology (KNUST)

We hypothesize that prevalence of phenotypic and indexed frailty will be >15% [13] and associated with older age, worse HIV control, female sex, greater social vulnerability and higher risk of death (VACS Index). For 151 PWH ≥ 50 years on stable antiretroviral therapy, we will assess physical frailty phenotype and frailty index [24,25], cognitive impairment, depression, social vulnerability [26], multimorbidity [27], polypharmacy and VACS Index–a measure of multi-organ function and physiologic frailty predictive of death [28–31].

#### Aim 2: Assess under-diagnosis and management of discrete cardiometabolic diseases in older PWH

We hypothesize that at 12 months, the proportion of positive screenings with: a) completed follow-up will be <50%; b) effective management of diabetes, hypertension, dyslipidemia, or renal dysfunction will be <50%. We will screen for hypertension, dyslipidemia, diabetes mellitus and renal dysfunction. The screening results will be compared to medical records and patient self-report to estimate rate of under-diagnosis and under-recognition. After sharing screening findings with participants, they will be referred to see their healthcare providers per the current practice. We will reassess them at month 6 and month 12 for: a) frequency and outcomes of follow-up (referral/treatment) for positive screenings; and b) progression and incidence of cardiometabolic diseases.

#### Aim 3: Identify barriers and facilitators to providing effective, patient centered healthcare to aging PWH

We will perform semi-structured qualitative interviews with up to: a) 30 PWH to assess engagement and satisfaction with healthcare for HIV, frailty and discrete diseases of aging; b) 20 HIV healthcare providers and policy makers to assess perceptions about caring for aging PWH; and c) 10 of our research staff about experiences conducting geriatric assessments in this study.

#### Aim 4: Capacity building in research skills that also have clinical relevance

We will hire research assistants who are new to or have minimal experience in scientific research and provide them training on how to conduct research. These trainings will be delivered through our weekly research meetings, and special research training seminars. Training areas will include; ethical conduct of research, administering informed consent, understanding of the research protocol, training on assessment procedures, and teaching on how to use project management tools such as Trello, Microsoft Outlook, and Redcap.

## Methods

### Study design

KAHO study is an observational study which will enroll 151 PWH aged 50 years and older from the KNUST Infectious Disease Unit (IDU). At enrollment, participants will complete the study assessment tools and provide biospecimens. Using data from the health assessments (Table 1), we will determine the prevalence of hypertension (systolic/diastolic blood pressure ≥140/90 mmHg on three occasions), dyslipidemia, dysglycemia, and renal dysfunction. We will use medical records assessment and participant self-report to estimate the frequency of under-diagnosis and under-recognition for these conditions respectively. These assessments will be used to create a referral list. The referral list will be shared with the participants and their healthcare team at the Infectious Disease Unit for further follow-up as indicated by existing standards of care. Participants will return at 6 and 12 months from baseline to repeat assessments and biospecimen collection. At each follow-up visit, the referral list will be updated to note new conditions and changes in management of existing conditions. We will assess the frequency of referrals made and completed. We will determine the proportion of conditions on the referral List for which a local, regional or national policy exists providing guidance on standards of care. For those conditions such as hypertension with existing standards, we will assess how closely the care provided adheres to the standard of care.

**Table 1:** Geriatric health assessments tools and instruments.

Domain	Description & Assessment (✓ = subjective assessment previously used in African setting)
Activities of daily living (ADL)	Daily routine self-care activities (e.g., eating, dressing, using the toilet) assessed by Katz ADL scale.✓ [32]
Instrumental activities of daily living (IADL)	Activities needed to live independently (e.g., cooking, managing finances) assessed by the Lawton IADL scale.✓ [32]
Lower extremity physical function	Balance, and lower limb strength assessed by Short Physical Performance Battery.✓ [32]
Fall risk	Screener: Ask “Have you fallen in the past year?” Confidence in doing 10 activities without falling assessed by Falls efficacy scale [33].
Upper extremity muscle strength	Assessed by Grip strength with hand dynamometer.✓ [32]
Multimorbidity	Disease screenings with evidence of moderate to substantial net benefit from screening for PWH [34] i.e., hypertension, tobacco or unhealthy alcohol or other drug use, hepatitis B and C, sexually transmitted infections, tuberculosis, diabetes mellitus, dyslipidemia, renal disease, VACS Index [28–31].
HIV	CD4+ T-cell count, HIV viral load, antiretroviral therapy regimen, AIDS defining conditions
Nutrition and weight	Mid-upper arm circumference, Skin folds, weight, height, and Mini Nutritional Assessment-SF ✓ [35,36]
Vision and hearing	Snellen Chart, Whispered voice test
Urinary/fecal continence	Screener: (1) In the past year, have you ever lost your urine/stool and gotten wet/soiled? and (2) If so, have you lost your urine/stool on at least six separate days? [37]
Pain	Pain Enjoyment, General Activity Scale for pain intensity and interference [38,39]
Polypharmacy	Use of four or more non-HIV medications, complimentary medications or supplements assessed by STOPP and START criteria for potentially inappropriate medication use in older adults.✓ [40,41]
Immunization	Record of vaccination for hepatitis B and COVID-19.
Falls	Fall Efficacy Scale [33]

We will conduct qualitative semi-structured in-depth interviews of PWH, healthcare providers at the Infectious Disease Unit and our study research assistants and technicians involved in conducting geriatric assessments. We will perform semi-structured qualitative interviews with up to: a) 30 PWH to assess engagement and satisfaction with healthcare for HIV, frailty and discrete diseases of aging; b) 20 HIV healthcare providers and policy makers to assess perceptions about caring for aging PWH; and c) 10 of our research staff about experiences conducting geriatric assessments in this study.

### Study setting

Kwame Nkrumah University of Science and Technology (KNUST) is an educational, scientific, and medical institution in Ghana and it is the institution managing this study. The Infectious Disease Unit of the KNUST Hospital will be the site for recruiting PWH. The Unit has about 1,800 registered PWH (>300 aged 50+years) and sees an average of 240 patients a month. KAHO KNUST study staff will conduct screening for eligibility. They will determine who to screen for eligibility using existing medical records of registered patients or patients who report for routine care at the clinic. Follow-up study visits will also occur at the clinic.

### Eligibility criteria

To be eligible to participate in the study, participants will need to be: a) HIV-positive; b) 50 years of age or older; c) Accessing prescribed highly active anti-retroviral therapy (HAART) for at least 6 months at the Infectious Disease Unit (IDU) of KNUST and d) able and willing to provide consent. Severely ill patients who cannot independently go through the study assessments will be excluded.

### Recruitment methods

Recruitment of participants for the study will include older PWH who have been routinely accessing treatment at the facility and will be identified through review of their medical chart. Review of their chart will be done by IDU staff (medical doctors, nurses, pharmacists, and laboratory staff) who are involved in the routine care of patients and will be trained and enrolled as study staff. Potential participants will be called on phone by the IDU nurse (study staff) and invited for screening at the IDU and possible recruitment into the study if they agree and provide informed consent. Other participants meeting the inclusion criteria will also be approached for possible recruitment when they report for routine clinic visit at the IDU.

Among the 151 study participants, we will purposefully select and interview 30 participants, 20 healthcare providers and policy makers, and 10 research staff over the duration of the study for the qualitative aim. Research assistants will help identify suitable and willing participants for these interviews using quantitative data from social vulnerability index assessment to sample people with higher and lower social vulnerability to increase the breadth of lived experiences to be discussed.

Recruitment of study participants is scheduled to start from 29^th^ January, 2024.

### Screening

Screening for the KAHO study will take place in two steps: 1) a pre-screen conducted through chart review and 2) in-person screening of participants who are eligible following the pre-screen. All potential participants will be assessed for eligibility based on the inclusion criteria.

A study nurse or research assistant will administer the full consent script for screening and obtain signed or thumb-printed informed consent from participants to participate in the screening. To ensure privacy and confidentiality, the names of participants will not be documented. Additionally, a KAHO study sticker will be placed on medical charts of patients who are screened or recruited to ensure that s/he is not approached again during the same visit. At each study visit, a study staff will search in the electronic study system for the hospital ID of an eligible patient to be sure that such a patient has not been previously enrolled in the study. This is to prevent double enrollment. To ensure privacy and confidentiality, the names of potential participants will not be documented.

### Visit flow

**Fig 1:**
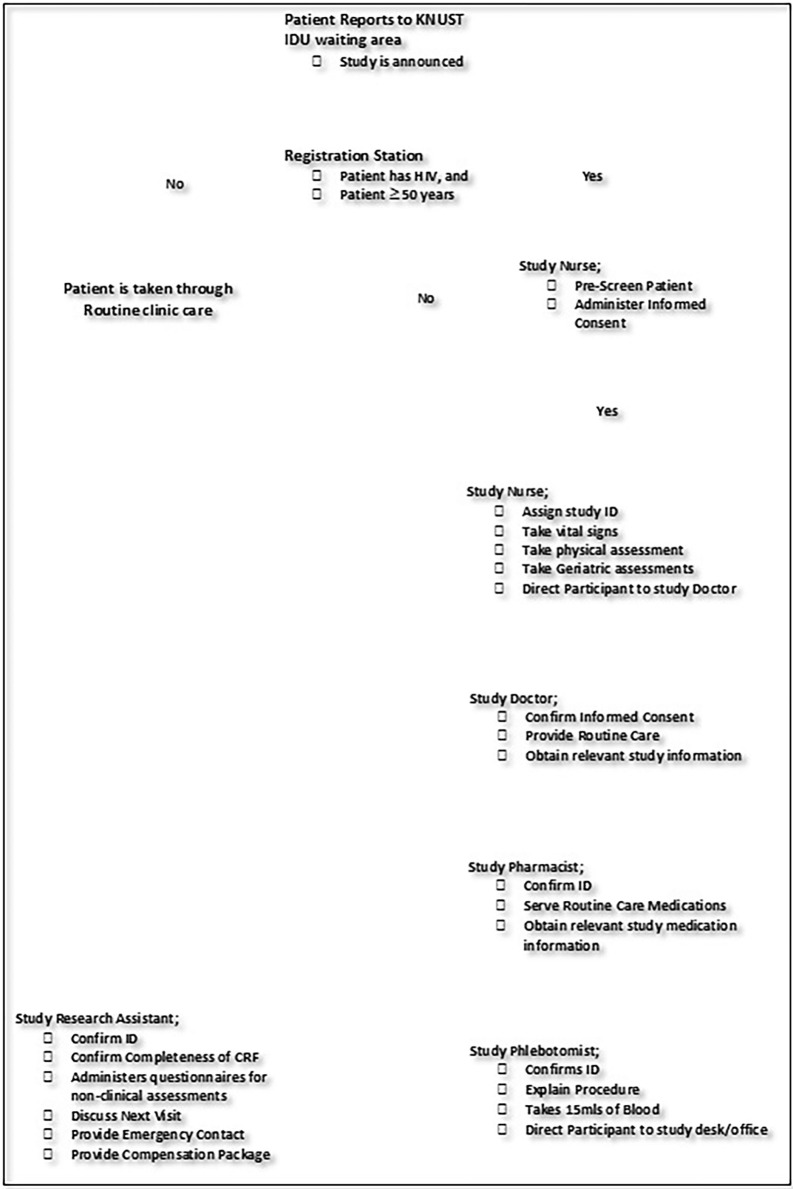
KAHO Study visit flow chart.

**Fig 1** below represents the major stages eligible participants will navigate during their study visits.

### Data collection

#### A. Chart review

Medical charts will be reviewed both prior to enrollment and after enrollment. Prior to enrollment, medical chart information about HIV status will be reviewed. We may review medical charts at the end of a participant’s participation in the study to monitor changes in medication regimens, treatment outcomes, as well as collect information from procedures that were part of routine clinical care.

#### B. Geriatric health assessments

Participants will undergo a focused geriatric health assessment covering vital signs and organ systems. These assessments will enable us to wholistically evaluate study participants [42]. In addition to the routine clinical examinations, we will pay particular attention to factors such as nutrition, vision, hearing, fecal and urinary continence, and polypharmacy (Table 1) [43].

#### C. Cognitive and mental health assessment

We will use the Mini-Cog tool which is composed of a clock drawing and a delayed three-word recall task to assess cognition including subtle impairments [44]. Mini-Cog is a tool designed to assess cognitive dysfunction, and has previously been used among the older people [45,46]. We will screen for depression using a two-question tool, “During the past month, have you been bothered by feelings of sadness, depression, or hopelessness?” and “Have you often been bothered by a lack of interest or pleasure in doing things?” [47] Participants will then complete a more comprehensive depression and anxiety scale (e.g., Geriatric Depression Scale) [48] and the Brief COPE [49] (measures coping strategies). These risk assessment tools have been used in Ghana and other countries in West Africa previously [49,50]. We will also administer the Patient Health Questionnaire-9 (PHQ-9) and General Anxiety Disorder questionnaire. Findings from the Geriatric Depression Scale will contribute to validation work of mental health assessment instruments previously used among Ghanaians; the PHQ-9 will be an additional dimension of mental health assessment in our study and its results will be compared with the former.

#### D. Social vulnerability

We will assess deficits in social support and engagement, living situation, self-esteem, sense of mastery or control over one’s life and socioeconomic status using a social vulnerability index assessment questionnaire [26,51]. This will provide information about positive and negative social experiences of aging with HIV in the cultural context of Ghana, and how these experiences facilitate or hinder ones recovery from other life stressors [52].

#### E. Blood and urine samples

We will collect 15mls of blood at each visit to assess for dyslipidemia (total cholesterol ≥ 5.2 mmol/l, LDL cholesterol ≥ 3.0 mmol/l, triglycerides ≥ 1.7 mmol/l, HDL cholesterol ≤ 1.45), Diabetes Mellitus (hemoglobin A1C ≥6.5) and renal dysfunction (eGFR < 60 ml/min), as well as CD4+ cell count and viral load, when required. We will also collect 5mls of urine for routine urine analysis.

### Data management

#### Sample size and power calculation

Our study is powered to detect whether the prevalence of phenotypic frailty is different from hypothesized 15% [53] With 126 people, 80% power at a 0.05 significance level, we can detect a minimum difference in prevalence of 10%. Assuming a Short Physical Performance Battery (SPPB; major component of frailty testing) mean score (standard deviation) of 10.2 (1.8) among West African PWH ≥50 years [32], 126 people would enable us detect a minimum difference in SPPB score of 0.25 in our sample compared to a reference population. This power is excellent given that minimum meaningful change estimates for SPPB score ranges from 0.4–1.5 [54]. We factored in 20% loss to follow-up to come to a final sample size of 151. We anticipate reaching theoretical saturation [55] from qualitative interviews with up to 30 patients, 20 healthcare providers and 10 research staff.

### Quality control and quality assurance

#### Assessment quality assurance

During the assessment, if the participant provides conflicting answers or answers that did not make logical sense (either within the same section or between sections), the RA will gently try to help the participant arrive at more logical answers. However, the RA will not force the participant to change his or her answers. The RA will review the self-administered section with the participant present. If many “refused” options are selected, the RA will offer the participant the opportunity to complete those sections (the RA will accept the participant’s refusal if he or she does not wish to complete the section).

The study will utilize paper-based case record forms and REDCap for data management. Data stored on computers will be password protected, and accessible only to research associates needing the information for follow-up purposes. Project data analyst will on a weekly basis, compare all paper forms with their associated electronic entry to assess accuracy. The RAs will be engaged weekly regarding any noted inconsistencies.

### Data analysis plan

#### Aim 1 analytic plan: Characterize frailty and multimorbidity in older PWH

**Frailty phenotype**: We will apply the Fried definition for physical frailty phenotype, which assesses components of unintentional weight loss, impaired grip strength and walking speed, fatigue and falling physical activity levels [6]. This definition categorizes people as frail if at least 3 out of the 5 components are positive for frailty. If 1 or 2 are components are positive, the definition categorizes them as pre-frail. If none, are positive, then frailty is considered absent. Prevalence of this frailty phenotype will be calculated as the proportion of participants who are not frail, pre-frail or frail. We will use a one sample proportion test to assess whether the proportion is significantly different from our hypothesized 15% for people who are frail. We will use multinomial logistic regression to identify correlates of the different frailty categories at baseline.

**Frailty Index**: For frailty assessed as an accumulation of deficits in health, we will create an index derived from 30–40 variables describing symptoms, signs, disabilities and diseases (see Table 1). These variables will be selected to meet five previously reported criteria [56]: 1) association with health status; 2) prevalence increases with age; 3) deficit effect should not saturate too early i.e., large proportion of population has deficit by relatively young age; 4) cover a range of health systems; and 5) same set of variables should be used for longitudinal assessments. To calculate each person’s index score, we will divide the number of deficits that apply to that individual by the total number of deficits in the index. Categorical variables will be coded to fit a scale of 0 (no deficit) to 1 (full expression of deficit) and fractional codes (e.g., a single intermediate response variable of maybe will be coded as 0.5 as it sits between a no deficit and full expression of the deficit). Continuous variables with thresholds related to a clinical hazard will be coded to fit on the 0 to 1 scale. For continuous variables without clinically accepted thresholds, a threshold will be selected that corresponds to a frailty index value of 0.2 when all other deficits are summed into the index score. The value 0.2 on the frailty index is recognized as approaching a frail state [57,58]. We will use linear regression to identify correlates of the frailty index score at baseline.

**Multi-morbidity and other analyses**: We will count the number of conditions co-morbid with HIV to estimate the prevalence and severity of multimorbidity. Additional analyses will examine the correlations between the two frailty measures and their association with multimorbidity, social vulnerability and 5-year mortality risk assessed by higher VACS Index Scores.

#### Aim 2 analytic plan: Diagnosis, recognition and management of discrete cardiometabolic diseases

Under-diagnosis of hypertension, dyslipidemia, diabetes mellitus, and renal dysfunction will be assessed as the proportion of existing conditions newly captured by screening during the current study. Under-recognition will be assessed as the proportion existing conditions that participants were not previously aware of. Positive screening follow-up rate will be assessed as the proportion of people with a positive screening that had a follow-up medical encounter related to that positive screening. We will re-assess blood pressure, lipids, hemoglobin A1c, and estimated glomerular filtration rate to determine disease progression among those with positive screenings and disease incidence among those with negative screenings. We will use a one sample test of proportion to assess whether the positive screening follow-up rate at 12 months is significantly different from our hypothesized 50%. To determine the correlates of disease management over time, we will use generalized linear mixed effects regression models [59]. Subject-specific random intercepts and slopes will be used to account for the correlation due to having repeated observations.

#### Aim 3: Barriers and facilitators to providing effective patient centered healthcare to aging PWH

Data analyses for this aim will be descriptive in nature; formal hypothesis testing will not be conducted. Qualitative interview data will be analyzed using the framework approach. This study aim will be guided by Levesque’s framework [60] of access to healthcare as a conceptual framework to identify both supply and demand-side factors that influence access to health care. A priori themes adapted from Levesque’s framework regarding engagement and satisfaction with healthcare for HIV, frailty and discrete diseases of aging and the impact of social vulnerabilities on their health as well as emerging themes from the data will form the basis of the framework analysis.

#### Aim 4: Capacity building in research skills that also have clinical relevance

Simple pre- and post-tests will be used to assess if there are any changes in the knowledge and skills of participants as a result of the sensitization and training sessions and capacity building activities. Thematic analysis of qualitative data from research staff interviews will be used to assess knowledge and skills gained and participant’s perception of the benefits and impact of the capacity gained.

### Ethics and dissemination

KAHO study has been approved by the Committee on Human Research, Publications and Ethics (CHRPE) KNUST with approval reference number CHRPE/AP/995/23, and Boston Medical Center with study IRB reference number H-44323.
